# Assessing Paramedic Confidence and Competence in Responding to Individuals with Neurodiversity: A Scoping Review

**DOI:** 10.1177/11786329251397296

**Published:** 2025-12-23

**Authors:** Kylie Kendrick, Georgina Seaman, Jodie McLellan, Alycia Jacob

**Affiliations:** 1 School of Nursing, Midwifery and Paramedicine, Australian Catholic University, Ballarat, VIC, Australia; 2 School of Nursing, Paramedicine and Health Sciences, Charles Sturt University, Albury, NSW, Australia

**Keywords:** paramedic, prehospital, neurodiverse, autism, education

## Abstract

**Objective:**

In this article, the findings from a scoping review are presented identifying what is known regarding paramedic confidence, competence and education in managing the neurodivergent patient.

**Methods:**

The Preferred Reporting Items for Systematic Reviews and Meta-analysis extension for scoping reviews (PRISMA-ScR) framework was used to identify what is known about the confidence, competence, preparedness and education when engaging with the neurodiverse patient.

**Results:**

Four articles were identified that explored paramedics, their confidence, competence and education in assessment and management of the neurodivergent patient, highlighting the need to increase knowledge and understanding pertaining to the neurodivergent patient in order to improve care.

**Conclusion:**

Given the unique role of the paramedic in contemporary health care, this study contributes to the discourse around appropriate levels of training for paramedics, as it highlights gaps in the literature associated with neurodiversity specific paramedic training.

## Introduction

### Awareness of Neurodiversity Is Increasing

Neurodiversity is a loosely defined term used to encompass a variety of neurodevelopmental conditions and learning disorders, including autism spectrum disorder.^1^ Following extensive review of the literature the authors have adopted the definition reported by Colombo-Dougovito et al^2^ which states: “*neurodiversity is an inclusive term for people on the spectrum of human attributes that affect their ability to contribute to presentations and applications across a broad range of social activities. They may be predisposed to respond in non-normative ways*”. The term neurodiverse will be used inclusively throughout this paper to describe people with Autism Spectrum Disorder (ASD), Aspergers, neurodiverse, neurodivergent and neuroatypical presentations. The term will exclude intellectual disability, acquired brain injury or mental illness associated with substance misuse. For the purpose of this study the authors have also adopted the terminology “the person with neurodiversity” acknowledging the person as having a lived experience of the condition. A global systematic review of prevalence studies by Zeidan et al^3^ has found that while rates vary significantly across contexts, the global prevalence of autism spectrum disorder diagnosis is around 1 in every 100 children The Australian Bureau of Statistics^4^ finding that 290 000 (1.1%) of Australians identified as living with ASD which is a 41.8% increase since 2018 demonstrating its increased recognition.

### Engagement in Healthcare Services

Awareness and diagnosis of neurodiversity is increasing globally, with a shift in health care and health promotion of neurodiverse persons towards a strengths-based approach.^5^ Contemporary research focuses on reducing stigmatisation of diagnosed conditions and supporting the movement towards a strengths-based approach, recognising individuals’ difficulty in responding to social activities in a normative way.^2,6^ People with neurodiversity may utilise pre-hospital emergency health services more frequently than other persons due to their unique health needs and vulnerabilities.^7,8^ This has led to an increased awareness of the need to focus on the importance of person-centred care extending beyond primary health care providers into the broader healthcare context.^9^ Healthcare providers have a diversity of exposure to education and training that reduces barriers to access.^10^ High sensory stimulus healthcare environments, communication challenges and fear of discrimination may exacerbate difficulties in accessing healthcare for people with neurodiversity, and increase their marginalisation and disadvantage.^11^

People with ASD, and other neurodiverse conditions have higher prevalence of co-occurrence physical conditions, than their neurotypical counterparts; statistically requiring healthcare engagement more frequently.^12^ Moreover, a data survey of Children’s health conducted between 2016 and 2019 identified that in children and adolescents between 12 and 17 rates of diagnosed depression were highest in persons with cooccurring autism and/or Attention Deficit /Hyperactivity Disorder (ADHD).^13^ Emerging research suggests that “autistic masking” can lead to a disconnect in identity which includes suppression of stimming and sensory suppression, this can be exhausting for people with neurodiversity, leading to increased suicidal ideation.^14^

### Neurodiversity in the Setting of Disaster Health

The neurodiverse population is often overlooked in traditional disaster and emergency management planning which historically has focused on physical disabilities. While there are a range of disability frameworks such as the Disability Access and Functional Needs Framework (DAFN), that do consider physical and intellectual disabilities, the specific needs of the neurodiverse persons are not well developed and should be considered outside of the term “disability”. Particularly in the setting of a strengths-based approach.

Disaster relief organisations are increasingly aware of the need to provide support to displaced or disrupted populations in a way that specifically includes awareness of population mental health. The neurodiverse population may have varying communication needs, be uncomfortable with crowded shelters, disruption of everyday routine, sensory overload from sirens, among other factors experienced during a disaster. There is evidence that first responders can improve their awareness of the specific needs of disabled people, when provided with targeted specific training.^15^ However, there is less awareness of first responder training for the needs of people with neurodiversity.

### Unique Role of Paramedics

Paramedics are often the first to engage with a person’s healthcare journey and do so as first responders with limited information about an individual’s background, medical history and individual needs from a biopsychosocial perspective.^16^ Although the term paramedic varies within the literature, this research has adopted the definition reported by Williams et al^17^ which was formed based on an International Delphi Study. Paramedics practice across a wide scope in varied healthcare settings and are uniquely placed to engage in first response, emergency and pre-clinical care. When providing care to a person with neurodiversity there are considerations that may be unique from other patient cohorts, such as the need to reduce stimulus or alter communication techniques.^11^ It is also important to acknowledge that some persons with neurodiversity may exhibit traits such as avoidance of eye contact that may contain different meanings than in other contexts.^10^

Considering this knowledge, it is imperative that paramedics can respond in an informed way to people with neurodiversity. Responding well requires knowledge, confidence and competence which can be learnt and taught on individual and organizational levels.

As such, the focus of this scoping review was to explore the paramedic preparedness when responding to the person with neurodiversity. Specifically:

(1) To determine what is known about the knowledge, confidence and competence of paramedics responding to the person with neurodiversity.
(2) Explore what, if any, education is provided to paramedics regarding the person with neurodiversity and reported on within academic literature.

## Methods

### Inclusion Exclusion Criteria

A structured search was undertaken in March 2023 to assess the available evidence related to the confidence, competence, preparedness, or education of paramedics in assessing and managing the person with neurodiversity. Studies were excluded if they focused on intellectual disability, acquired brain injury or mental illness associated with substance misuse. No date limit was set.

### Study Design

Scoping reviews are ideal for addressing a broad research question in order to: identify the body of literature on a subject matter; identify knowledge gaps; and commonly a precursor to a systematic review.^18^ As the research profile, and evidence of paramedicine is evolving, a scoping review was deemed appropriate to support the research question and a standardised approach to identifying literature and its examination. This scoping review utilised the principles reported in the Preferred Reporting Items for Systematic Reviews and Meta-analysis extension for scoping reviews (PRISMA-ScR).^19^ This framework involves: identifying the research question; identifying relevant studies, study selection, data charting; reporting of the results.^19^

### Search Strategy

Key words from the research question were utilised to inform the search strategy as outlined in Table 1.

**Table 1.** Key Words and Concepts.

Neurodiversity	Education (including knowledge, confidence and competence)	Paramedic
“Neurodiv*” OR “autis*” OR “asperger*” OR “neuroatypical” OR “neurodevelopment” OR “spectrum”	“Confiden*” OR “skill” OR “study” OR “learn*” OR “require*” OR “prepare*” OR “read*” OR “understand*” OR “knowledge” OR “qualif*” OR “professional development” OR “education” OR “accredit*” OR “competen*” OR “train*”	“Paramedic*” OR “emergency” OR “pre-hospital” OR “EMS” OR “EMT” OR “ambulance” OR “emergency medical technician*” OR “out-of-hospital”.

### Information Sources

Databases searched included CINAHL, Embase, ERIC, Global Health, MEDLINE and PsycInfo. No limits were placed on the publication dates. To be included the studies were required to be in English, primary research and peer reviewed. Case reports, abstracts, editorials and grey literature were excluded.

### Selection and Data Collection Process

All articles were downloaded into Endnote,^20^ with duplicates identified and removed, prior to being uploaded to the systematic review management program Covidence.^21^ The title and abstracts of all studies were screened by at least two authors based on the inclusion and exclusion criteria, and any article that did not meet the criteria was excluded. Any articles that met the criteria, or where it was unclear if they met the criteria from the abstract, were obtained for full review. The full text of each paper was accessed for assessment by at least two authors. Any further articles that did not clearly meet the criteria at this point were excluded. Any reviewer conflict that arose was reviewed by a third author. The reference list of each of the included studies was also hand searched, although no further studies were identified.

### Data Extraction

Data were extracted by two authors, in Covidence using a bespoke data extraction tool with discrepancies being reviewed by all four authors collaboratively. The data extracted included author/s; year; country; aim of study; study design; recruitment method; study length; funding sources; terms used for paramedic; groups of neurodiverse people considered; education materials used; educational concepts considered; outcomes; outcome measures and validated tools utilised for data collection. The authors undertook discussions to identify coding that applied to the identified results. The authorship team included people with lived experience of neurodiversity and ambulance services, allowing them to confidently identify the elements of neurodiversity and educational constructs set out in the literature.

### Quality Assessment

Studies were assessed for quality and risk of bias using the Mixed methods appraisal tool (MMAT)^22^ This tool was specifically designed in 2006 for the appraisal of mixed study reviews within five categories (qualitative research; randomised control trials; non-randomised studies; quantitative descriptive; and mixed method studies). There is no overall score applied to a study, however the detailed presentation of the quality allows for a clear comparison of studies where contrasting of results is undertaken.^22^ The MMAT tool was utilised within the Covidence systematic review platform, where two reviewers completed quality assessment independently. Any discrepancies were reviewed by all four authors collaboratively.

## Results

### Selection of the Studies

**Figure 1.**
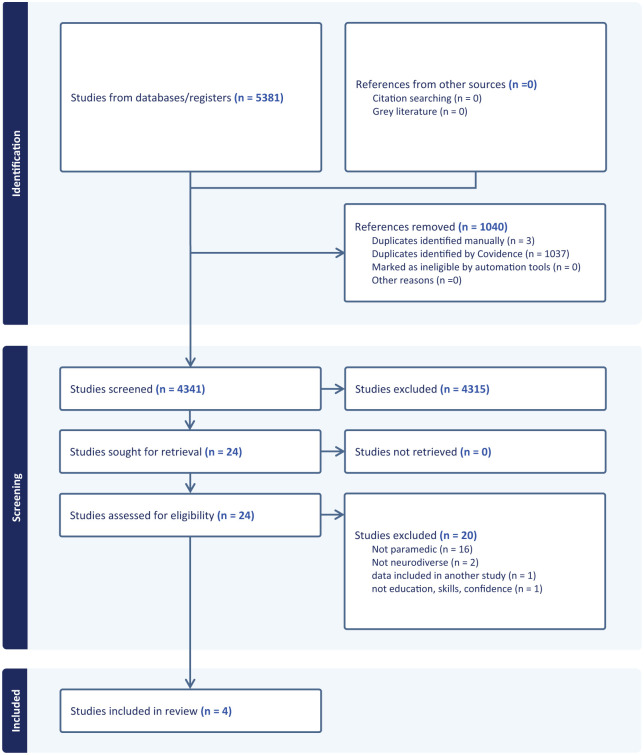
PRISMA flowchart of included studies.

The structured search found 5381 articles, and of these 1040 duplicates were identified and removed, leaving 4341 studies. Four thousand, three hundred and fifteen studies were excluded based on the title and abstract; and twenty-six articles were retained for full text review. Of the 26 articles reviewed at full text, a further 22 were discarded due to not meeting the inclusion criteria. The final sample included four articles.^16,23-25^ Article selection is presented in Figure 1.

### Description of the Studies

A total of four articles were extracted and are presented in this scoping review. All the studies originated from the United States and were undertaken between 2014 and 2020, and all included Autism as the primary neurodiverse condition. Three of the studies were quantitative in nature and were survey based,^23-25^ with the remaining presenting qualitative data only.^16^

All of the studies included paramedic knowledge, three considered confidence^16,24,25^ and two considered competence.^23,24^ Paramedic education and training was assessed in three of the four papers.^23-25^ The level of evidence in all four papers was rated moderate when quality assessed.^22^

### Neurodivergent People Considered

Whilst the term neurodiversity was explored by researchers to facilitate a broader examination of presentations, articles only on Autism Spectrum Disorder were found. All studies framed the conversation and training requirements of first responders with persons who have Autism as an adjustment framing with a deficit-framed narrative. One study (Kelly) assessed the ASD awareness of first responders who participated in mandatory training but designed the survey to assess the adequacy of training in recognition and awareness of “disabled citizens”, and “special needs service users”. Love et al^24^ assessed the effect of training on recognition of, and strategies to manage people living with ASD specifically. Similarly, McGonigle et al^25^ assessed the effectiveness of ASD specific educational materials whilst Wachob and Pesci^16^ assed the confidence and knowledge of emergency service personnel with individuals with ASD.

Despite all studies originating from the USA, there was no consistency between the educational materials provided to paramedics, nor tools to evaluate their implementation. One study reported using the Emergency Network Autism Community Training (ENACT) which comprised of direct lectures, paired discussions, video clips, and open discussions including and question and answer period,^24^ two other studies utilised training manuals, DVD/video-based training, inclusive of internet-based training.^23,25^ The final study did not include its own educational component, however it did report that autism specific training was associated with an increased level of comfort in paramedics, but no associated knowledge increase based on the Western Region ASERT Autism Spectrum Disorder Survey.^16^

Three studies reported on the impact of autism specific training, one study identified that self-rated knowledge, confidence and comfort improved significantly (*P* < .001) after training,^24^ another identified a statistically significant change in 20 out of 25 markers following completion of the ASERT program.^25^ Whilst Wachob and Pesci^16^ did not provide statistics, however, they identified that previous autism – specific training was associated with increased comfort levels, however had no significant impact on knowledge levels regarding autism. The final study identified that a significant portion of participants had not undertaken state mandated training related to autism, recommended within New Jersey, and of those that did, 46% reported feeling that the training was only somewhat or not effective and 68% identified no training or follow-up was available.^23^ Nil studies reported on the use of persons with neurodiversity or patient advocates in the design and implementation of any training materials, although this may have occurred (Table 2).

**Table 2.** Study Selection and Characteristics/Data Extraction Chart.

Author, year, country	Aim of study	Study design	Participants and neurodiverse groups considered	Educational materials used	Educational concepts and how they were measured	What were the outcomes
Love, 2020, United States	Evaluate the initial effectiveness of ENACT (Emergency Network Autism Community Training)	Pre post intervention survey part of in-service professional development	224 diverse health professionals 1 paramedic Focused on general ASD	ENACT (Emergency Network Autism Community Training) Direct lecture, paired discussion, video clips, Q &A and open discussion	Knowledge; confidence; competence Other: comfort, quality of training Survey tool adapted from Autism Stigma and Knowledge Questionnaire (ASK-Q)	Self rated knowledge improved significantly *P* < .001 pre M = 2.75, SD = 1.23, post M = 4.17, SD = 0.66 Self rated confidence improved significantly *P* = .001 pre M = 3.59, SD = 1.05, post M = 4.21, SD = 0.75 Self rated comfort significantly improved *P* < .001 pre M = 3.66, SD = 1.07, post M = 4.24, SD = 0.72 Directly addressed the gap in the literature related to specific training for first responders. Results highlight the need to provide proactive solutions to prevent negative interactions between first responders and people with neurodiversity
Kelly, 2016, United States	Evaluate the level of training regarding autism awareness among emergency service personnel in the State of New Jersey	Cross sectional study Funding from Kean University Faculty Summer Research Program and the National Institute on Drug Abuse of the National Institutes of Health	(222 participants 57 paid and unpaid EMS Focused on adults and children	Assessed existing training including 1. read and sign 2. video based training 3. internet based training 4. speaker/instructor 5. combination	Knowledge; preparedness; competence; unvalidated survey tool	New Jersey jurisdiction mandates autism and hidden disability recognition and response training. 46% felt training was only somewhat or not effective and 68% stated no follow up training or refreshers were available
McGonigle, 2014, United States	To develop structured didactic materials and training sessions for emergency medical services and emergency department personnel on ASD and to evaluate their effectiveness in imparting knowledge on autism spectrum disorders and improving the subjective comfort and awareness of these health care providers on how to aid ASD patients in crisis situations	Case report recruited conference participants at emergency medicine conferences and professional networks Funding from Bureau of Autism Services, Department of Public Welfare, Commonwealth of Pennsylvania to the Western Regional Autism Services, Education, Resources, Training (ASERT) Collaborative at the Western Psychiatric Institute and Clinic of UPMC. No conflict of interest declared	110 (made up of 38.2% Emergency nurse, 7.3% Paramedic, 46.3% EMT, 8.2% Other including Student) Focused on adults with autism	A training manual and DVD with dramatised case examples and commentary from individuals with ASD and their family members. separate training manuals and DVDs were created for prehospital and emergency departments	Knowledge; confidence; comfort; self-reported survey, unvalidated tool	Statistically significant change in 20/25 markers
Wachob, 2017, United States	To determine the knowledge and confidence of EMS personnel on interacting and treating an individual with ASD	Qualitative research recruited professional networks EMT and paramedics working in six counties across Western Pennsylvania No funding or conflicts of interest declared	73 participants EMT (n = 28), and paramedics (n = 45). Focused on adults and children with autism	N/A	Knowledge; preparedness; confidence Data from larger survey – unclear if tool was validated. Western Region ASERT Autism Spectrum Disorder Survey	Autism-specific training was associated with increased comfort levels but had no significant impact on knowledge about ASD. Efforts to ensure emergency responders are properly prepared should also be further investigated. Familiarity with autism was found to significantly influence knowledge and comfort scores. (1) demo-graphic characteristics were associated with increased knowledge and comfort; (2) having autism-specific training and having autism-specific resources were the two lowest scored items; and (3) interactions with individuals with autism increased both knowledge and comfort in emergency responders.

## Discussion

This scoping review aimed to explore the confidence, competence and education of paramedics in responding to people with neurodiversity. Our results highlight the inconsistency of training outcomes to improve paramedics confidence in managing the person who is neurodiverse. The results of this study have indicated that knowledge, confidence and competence are not always achieved through increased training materials with mixed results in accomplishment of these outcomes when specifically managing persons living with neurodiversity. The studies revealed previous personal experience with people with Autism as positively correlated with knowledge and comfort.^16,25^ This compared with results from training methods used in the studies which revealed mixed results in improvement of knowledge, confidence and competence when didactic training materials were used.^16,23-25^

### Understanding the Diversity of Neurodiversity

Although the umbrella term neurodiversity and its included sub terms were utilised in the search, literature focused on individuals with ASD. Historically, autism has been seen as inherently disabling and widely associated with negative behaviours that may have resulted in higher levels of caution for services interacting with people living with an autism diagnosis.^26^ Increasingly, there is community awareness that the term “neurodiversity” better recognises the strength-based approach to validating the developmental difference rather than disablement of ASD. Additionally, the term better recognises the encompassing of other neurodivergences like learning or developmental disabilities, ADHD, obsessive compulsive disorders, communication disorders and other neurological minorities. Without reducing the specificity of education materials to prepare paramedics when treating people with neurodivergence, the term enables collation of materials and resources to support agency in clinician’s confidence, competence and education around neurodiversity.^27^ Educational material for paramedics must reflect the growing need to recognise individuality over the education of neuro-normative clinician perspective.

### Training Materials

Experiential learning is a known method to improve confidence and competence for “learners” by situating their learning in context, although beyond of the scope of this review. In our study 2 studies incorporated aspects of experiential learning. When analysing this data through an “experiential lens”, it is evident to see how learners who are involved and active participants alongside persons with neurodiversity, are able to situate their knowledge in place and time.^28^ However, due to the dynamic work environment of pre-hospital care, the paramedic role demands translation and inquiry into real-world problems. Learners must be exposed to the novel experiences alongside people who are neurodiverse to facilitate critical reflection that mediates learning.^28,29^ Although limited by the breadth of findings revealed from this study, there is evidence of need for further focused research that identifies the value in experiential learning to facilitate confidence, competence and knowledge. There is great opportunity to situate learning for the paramedic in a contextual environment that focuses on achieving improved interpersonal communication skills that facilitates interactions with persons living with neurodiversity.

### Education Focus

Since people with neurodiversity may experience and respond differently to different or unexpectedly to stimuli and medical examinations, it is imperative that paramedics are able to recognise signs that an individual experiences neurodiversity and alter care and communication accordingly. Literature which has examined the training required to develop the confidence of other healthcare professionals in helping to treat medical needs for people living with neurodiversity has identified the need to focus on interpersonal skills development.^30^ Staff experiences identified the need for development of skills that improve communication, as well as behaviour and mental health management to provide person centred care.^30^ Not only is this reported from providers, but additionally individuals with neurodiversity and their families report communication as a significant barrier for patients to participate in assessment and treatment.^31^ Interpersonal communication skills are most constructively developed through practice opportunities^32^ and further support the rationale for research investigating paramedic development of these skills alongside practical interprofessional learning opportunities.

### Strengths Based Approach

The breadth of literature on neurodiversity has been challenged by a reframing of specifically “autism spectrum disorder”, to a strengths-based discourse around neurodiversity. Despite the conventional medical paradigm framing “Neurodiversity as a disability”, not only does this narrative require systemic change, but so too does our perspective on training for addressing the needs of neurodiverse persons.^33^ Sustained attempts to educate paramedics on neurodiversity may continue to yield unsuccessful results without improved recognition of the role individuals living with neurodiversity can play in outlining their own needs. People living with neurodiversity can be engaged in development of materials which construct knowledge, confidence and competence in paramedics when attending their presentations in out-of-hospital environments. The medical model is largely “individualist” and risks dismissing opportunities to learn from the social and environmental context which situates presentations to paramedics (emergency services/ambulance services etc.).^33^ The development of resources and research has primarily arisen from increased healthcare demand in the US. An Autism Healthcare Accommodations Tool (AHAT) developed by Academic Autistic Spectrum Partnership in Research and Education (AASPIRE) in the USA includes a range of strategies that healthcare workers can easily adopt in communication with people with Autism.^34^ When utilised, the resource has shown to improve self-efficacy and patient provider communication, and it’s success is largely attributed to the co-design between consumer, researcher and healthcare provider.^35^

### Recommendations

- Further study should be conducted to identify the specific competency requirements for paramedics when engaging with neurodiverse communities, from the perspectives of both paramedics and people with neurodiversity.
- Education to support paramedics to provide care for neurodiverse people should be co-designed with neurodiverse communities and implemented in a structured curriculum, reducing ad hoc reliance on personal experience.
- While training methods should be chosen based on considerations of the individual services and their learning goals, key competencies could be embedded in registration requirements to ensure standardised levels of knowledge.
- Further studies should also consider the effectiveness of educational programs, including using outcomes identified at user and service level.

### Strengths and Limitations

This study identified several methodological limitations across the reviewed studies which has potential to impact interpretation of the results. First the sample sizes explored in studies was small and therefore limits the generalisability of the findings. The level of evidence was also moderate, and no randomised controlled trials were included. The lack of quantitative, interventional and longitudinal studies that have utilised mixed methods of understanding training and education around neurodiversity in Paramedicine also limits the generalisability of findings. The lack of published literature in Paramedicine limited the scope of research that may have been included and there is a possibility that literature in other disciplines would serve value in indicating training success. In addition, grey literature was not examined, and the study potentially failed to incorporate some relevant literature. The study however has several strengths including the use of a strong methodology and strategy with the use of international sources, the authors maintained a broad consideration of what it means to have knowledge, competence and confidence, rather than reviewing these in isolations which has previously been done within other research. Additionally, the authors considered neurodiversity as an umbrella term for a range of conditions, and has taken a strengths-based approach, with person centric focus to the recommendations reported.

## Conclusion

Paramedics have a unique role that often places them as the first healthcare response for people living with neurodiversity accessing healthcare systems. In cases where education and training materials have been reported on, clinician confidence and competence has been associated with lived experiences with people with neurodivergence. The association between education materials and clinician confidence and competence was not demonstrated within the literature found and more research on successful training should be carried out. There is limited evidence on the ability of paramedics to recognise and respond to their needs in a condition specific way, and little evidence on education provided in this space. Further research in this area is warranted to support holistic person-centred care.
